# Quantification of heterogeneity in lung disease with image-based pulmonary function testing

**DOI:** 10.1038/srep29438

**Published:** 2016-07-27

**Authors:** Charlene S. Stahr, Chaminda R. Samarage, Martin Donnelley, Nigel Farrow, Kaye S. Morgan, Graeme Zosky, Richard C. Boucher, Karen K. W. Siu, Marcus A. Mall, David W. Parsons, Stephen Dubsky, Andreas Fouras

**Affiliations:** 1 Department of Mechanical & Aerospace Engineering, Monash University, Melbourne, VIC, Australia; 2 4Dx Limited, Melbourne, VIC, Australia; 3 Robinson Research Institute, University of Adelaide, SA, Australia; 4 Women’s and Children’s Hospital, North Adelaide, SA, Australia; 5 School of Physics and Astronomy, Monash University, Melbourne, VIC, Australia; 6 School of Medicine, University of Tasmania, Hobart, TAS, Australia; 7 Department of Medicine, University of North Carolina at Chapel Hill, Chapel Hill, NC, USA; 8 Department of Translational Pulmonology Translational Lung Research Center (TLRC), Member of the German Center for Lung Research (DZL), University of Heidelberg, Heidelberg, Germany

## Abstract

Computed tomography (CT) and spirometry are the mainstays of clinical pulmonary assessment. Spirometry is effort dependent and only provides a single global measure that is insensitive for regional disease, and as such, poor for capturing the early onset of lung disease, especially patchy disease such as cystic fibrosis lung disease. CT sensitively measures change in structure associated with advanced lung disease. However, obstructions in the peripheral airways and early onset of lung stiffening are often difficult to detect. Furthermore, CT imaging poses a radiation risk, particularly for young children, and dose reduction tends to result in reduced resolution. Here, we apply a series of lung tissue motion analyses, to achieve regional pulmonary function assessment in β-ENaC-overexpressing mice, a well-established model of lung disease. The expiratory time constants of regional airflows in the segmented airway tree were quantified as a measure of regional lung function. Our results showed marked heterogeneous lung function in β-ENaC-Tg mice compared to wild-type littermate controls; identified locations of airway obstruction, and quantified regions of bimodal airway resistance demonstrating lung compensation. These results demonstrate the applicability of regional lung function derived from lung motion as an effective alternative respiratory diagnostic tool.

The assessment of lung function is critical in the diagnosis of lung diseases such as asthma, chronic obstructive pulmonary disease (COPD) and cystic fibrosis (CF). Structural changes in the lungs of patients with lung disease alter the flow of air and motion in the lungs, whether by obstruction, increasing airway resistance, or by changes to the lung parenchyma which alter the mechanical properties of the tissue. Current clinical assessments and treatments of lung disease are indirect and since many treatments have adverse side effects, they cannot be applied prophylactically. Therefore, earlier and more accurate disease diagnosis are keys to successful intervention.

Changes in lung function are typically tracked using spirometry with measures such as forced expiratory volume in 1-second (FEV_1_) and forced vital capacity (FVC) being widely used to quantify airway obstruction. However measures from spirometry provide no structural information^1^,^2^,^3^, are global measures of lung function and as a result cannot capture the heterogeneity in lung diseases such as COPD and CF. As FEV_1_ is effort dependent it is often a difficult test to perform in young children, the group where preventative measures should be applied. The forced oscillation technique (FOT) is a non-invasive method for assessing the resistive properties of the respiratory system^4^,^5^. FOT is effort independent, meaning that this test can be performed on children, the elderly, and ventilated patients. Furthermore, one of the more notable advantages of FOT over spirometry is its sensitivity to detecting peripheral airway obstructions^6^. However, clinical assessments using spirometry or FOT yield a global measure of lung function, thus concealing variations in lung disease, especially in cases with functional compensation from surrounding healthy lung tissue. Recently, multiple breath gas washout (MBW)^7^,^8^ measures such as lung clearance index (LCI) have also been shown to be more sensitive than FEV_1_ for assessing lung function in early stage lung disease^9^.

Imaging techniques such as conventional radiography and computed tomography (CT) continue to be used to profile lung disease. CT is increasingly used to evaluate lung health in humans^10^,^11^,^12^ and in small animal studies^13^. As CT provides excellent structural information, experienced radiologists are capable of identifying regions of the lung that are poorly aerated, as well as airways affected by mucus plugging. However, measures of lung stiffness and ventilation are not directly available, with the key surrogate being lung aeration. CT scoring systems such as CF-CT and PRAGMA-CF are currently used for quantifying the extent of lung disease at various stages^11^,^14^. Unsurprisingly, CT has become increasingly popular as an outcome measure to assess therapeutic efficacy and although there are questions about the danger of radiation from CT^12^,^15^, it is generally accepted there is increased cancer risk from repeated radiation exposures. Magnetic resonance imaging (MRI) is an alternative to current X-ray based imaging modalities but its utility has not been exported to the general community^16^,^17^.

Phase contrast X-ray imaging (PCXI) is particularly suited to imaging lung dynamics^18^,^19^,^20^ since the air-tissue interfaces between the air sacs greatly increase image contrast. In this study, we combine PCXI with a volumetric particle image velocimetry (4DPIV) image processing technique to obtain highly sensitive measures of lung tissue expansion, or local change in lung volume. Local lung tissue expansion (volume change) is utilised here to assess the functional capacity of lung tissue as it is physiologically proportional to regional ventilation. Using an airway tree linking process (ATL) we associate lung tissue ventilation with a segmented airway tree^21^, allowing the calculation of time-resolved regional airflow. Since lung disease alters airflow, this approach provides a sensitive technique for detecting, localising and quantifying regional lung function deficits. The aim of this study was to illustrate the possibilities of imaging altered lung motion to quantify the heterogeneous nature and subsequent phenotyping of lung disease. Here, we quantify the heterogeneity of lung function in live β-ENaC-Tg mice, a well-established model with CF-like lung disease^22^,^23^. Overexpression of the epithelial Na^+^ channel (ENaC) in the conducting airways causes airway surface liquid depletion and increased mucus concentration that produces reduced mucus clearance, airway mucus obstruction, chronic neutrophilic inflammation, structural lung damage and substantial pulmonary mortality^24^ all characteristic features of CF lung disease in humans.

## Results

### Imaging lung motion yields insight into regional airflow within the lungs

**Figure 1:**
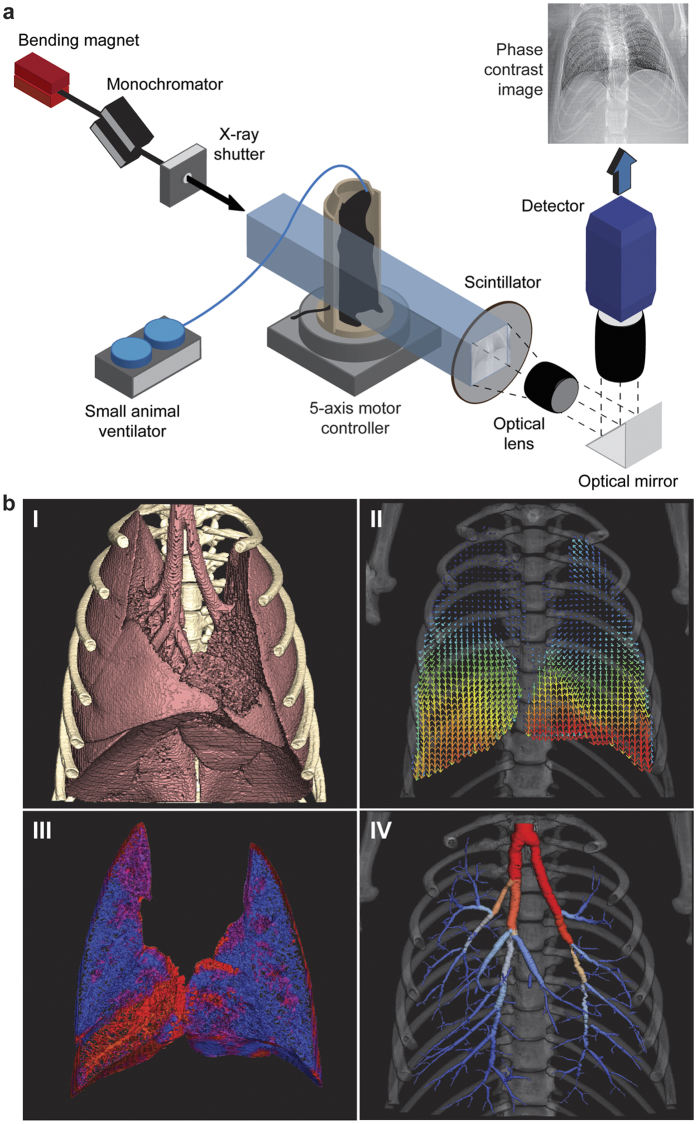
Synchrotron experimental setup and data processing sequence. (**a**) A simplified schematic of the PCXI experimental setup is shown. The mouse is mechanically ventilated and imaged while rotated using a 5-axis motor controller (Aerotech, USA). (**b**) (I) The raw image data is binned against the ventilation trace, pre-processed and reconstructed. (II) 4DCT imaging followed by lung motion image processing yields a 4D map of the local displacement (blue = small displacement; red = larger displacement) of lung tissue between successive CT volumes. Only data at peak inspiration shown here. (III) Inter-volume displacement gradients are used to determine time-resolved regional ventilation (red = low ventilation, blue = higher ventilation). (IV) Using the ATL process, time-resolved regional ventilation measures are associated with the segmented airway tree from CT to obtain time-resolved regional airflow (airway tree segment colouring).

Four-dimensional computed tomography (4DCT) utilising phase contrast imaging (Fig. 1a) in live anaesthetised and intubated mice under mechanical ventilation produced images that were processed and analysed (see Methods) to provide local measures of airflow in the airway trees at 16 time points throughout the (0.45 seconds) respiratory cycle (Fig. 1b). Using 4DPIV, the lung tissue motion can be measured (Fig. 1b. II) from which local lung tissue expansion is directly calculated (Fig. 1b. III). The airway tree, segmented from a single CT reconstruction, is associated with ventilation to determine airflow at each terminal airway tree point, or ‘endpoint’ (see Methods). In this study, these endpoints are the most distal branches of the tree that could be digitally segmented. Local measures of airflow at each endpoint are recursively summed up the tree to determine regional airflow at each segment of the airway structure (Fig. 1b. IV).

### Global pulmonary function measures are limited

**Figure 2:**
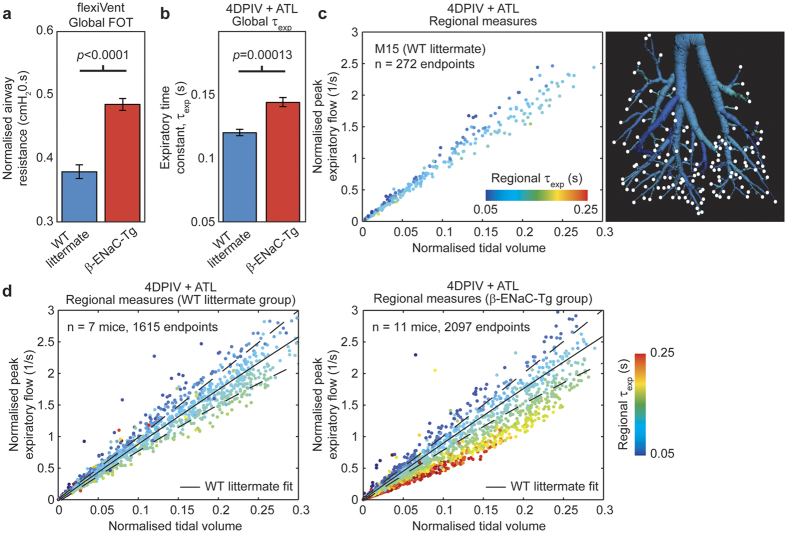
Global pulmonary function measures provide limited information. (**a**) Physiological lung function measures (flexiVent) of wild-type littermate controls (WT) versus β-ENaC overexpressing (β-ENaC-Tg) mice validates the regional pulmonary function method presented here. β-ENaC-Tg mice showed significantly increased specific airway resistance compared against littermates. While airway resistance was measured using a global forced oscillation measure, the dynamics of the lung tissue and the spatial distribution of disease are not captured by FOT. (**b**) Averaged global expiratory time constant, τ_exp_, measures in WT littermate and β-ENaC-Tg mice from 4DCT and lung motion analysis (4DPIV) followed by regional calculation of airflow using our ATL method. (**c**) Scatterplot of normalised local peak expiratory flow against normalised tidal volume at endpoints in the segmented airway tree of a single representative WT littermate mouse (M15). Each point on the scatterplot corresponds to each endpoint in the airway tree (white dots in adjacent airway tree image) and are coloured by the local expiratory time constant calculated using the regional lung motion data. (**d**) Scatterplot of normalised local peak expiratory flow against normalised tidal volume for endpoints in all WT littermate mice (left) and β-ENaC-Tg mice (right) used in this study. The scatter points are coloured by local expiratory time constant calculated using the methods discussed in this paper. The polynomial fit (solid lines: fit, dashed lines: 99% confidence interval limits) to WT littermate data (black lines shown in both panels) illustrates the effect of lung disease on lung function in β-ENaC-Tg mice.

Physiological measures obtained from flexiVent-derived forced-oscillation pulmonary function testing (FOT) manoeuvres for normalised airway resistance of WT littermate controls versus β-ENaC-Tg mice are shown in Fig. 2a. To produce a surrogate for a global measure of lung function from the imaging data, regional airflows in each segment of the airway tree were recursively summed from the endpoints up the airway tree to the trachea. This allowed for the global expiratory time constant, τ_exp_, to be calculated (Fig. 2b). Both FOT and imaging through measures for airway resistance and expiratory time constant respectively, show the effect of obstructive lung disease on airflow resistance. However, as these analyses are global, the spatial distribution of lung disease is not fully captured by either FOT measures for airway resistance or our surrogate for a global measure, the global expiratory time constant.

Quantification of regional airflows along the airway trees allows for a detailed insight in to the regional nature of lung function. Fig. 2c shows the normalised peak expiratory flow (normalised to local tidal volume at each endpoint) plotted against normalised tidal volume at each endpoint for a single healthy WT littermate mouse (M15). Each point represents local airflow data at each endpoint in the airway tree (white dots in adjacent airway tree image) and is coloured by local expiratory time constant. When considering the two groups of mice in this study (Fig. 2d) the wealth of information available from assessing regional lung function is evident. β-ENaC-Tg mice (Fig. 2d right panel) show a significant regional decrease (*p* < 0.001 between polynomial fits to WT littermate and β-ENaC-Tg groups, see Supplementary Fig. 1a) and delay in the ventilation of lung tissue during the respiratory cycle (resulting in increased local expiratory time constant) in contrast to WT littermates (Fig. 2d left panel). The data also shows regions of lung (in β-ENaC-Tg mice) with increased ventilation (see Fig. 2d left panel and Supplementary Fig. 1b) indicating signs of lung compensation to maintain overall lung function. Fig. 2d shows that the β-ENaC-Tg mice produce lower peak expiratory flows and longer expiratory delays in peripheral airways in contrast to WT littermates.

### Functional imaging shows the heterogeneity of patchy lung disease in β-ENaC-Tg mice

**Figure 3:**
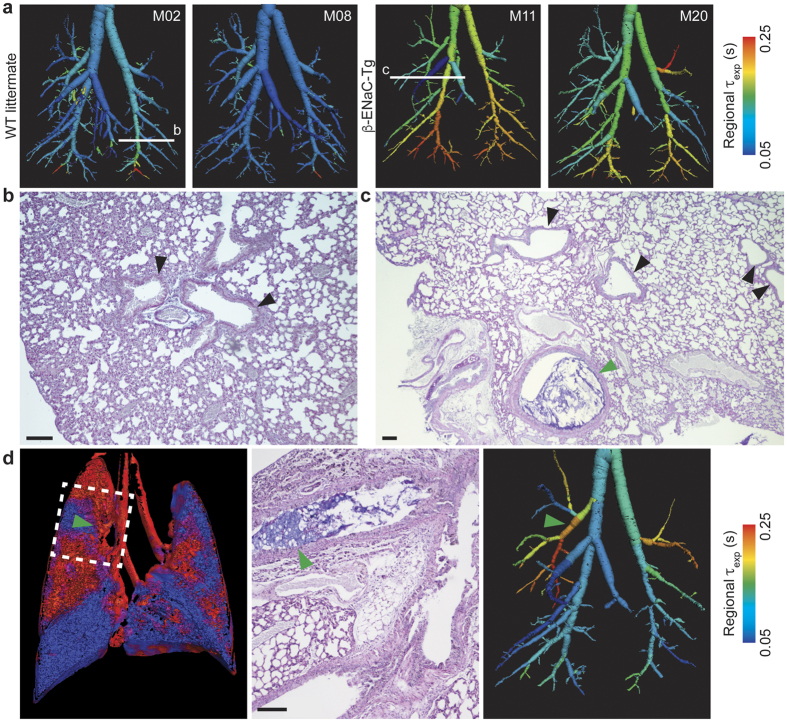
Functional imaging quantifies the extent to which β-ENaC-Tg mice express patchy lung disease. (**a**) 4DPIV followed by ATL (coloured by expiratory time constant) show that lung disease in β-ENaC-Tg mice is heterogeneous. Histological sections of the lung are obtained at different locations (white lines) and are shown in (**b**,**c)**. (**b**) AB/PAS stained histological sections from the same WT littermate as 3a (M02) shows no mucus plugging (black arrows). (**c**) AB/PAS stained histological sections from the same β-ENaC-Tg mouse as 3a (M11) shows clear airways (black arrows) as well as mucus obstructed airways (green arrow) within the same lobe, indicating non-homogenous disease. (**d**) Regional lung ventilation in β-ENaC-Tg mouse (M22) shows low ventilation in the right upper lobe of the lung (left panel, dashed box). (Middle panel) The corresponding histological section showing mucus blockage in the bronchial tree that feeds the right upper lobe of the lung. (Right panel) Airway tree coloured by expiratory time constant, the same analysis applied to data in a, shows the right upper region of the lung exhibits an increased resistance. This is validated by the mucus blockage evident in histology shown in the middle panel. Green arrow shows location of a mucus obstruction. Scale bars, 100 μm.

Segmented airway trees (Fig. 3a) exhibit the heterogeneity of CF-like lung disease in β-ENaC-Tg mice, as evidenced by patchy regions of increased expiratory time constants. Typical AB/PAS stained histological lung sections from a WT littermate mouse (Fig. 3b) and a β-ENaC-Tg mouse (Fig. 3c) show that not all βENaC airways exhibit mucus blockages, consistent with heterogeneous lung disease. Regional tissue ventilation, subsequent time constant mapping, and matching histology showcase mucus obstruction detected in the right upper lobe of the β-ENaC-Tg mouse (Fig. 3d, green arrows). Our analysis revealed mucus blockages restrict airflow, thus increasing the local time constant, leading to regions in the lung with low ventilation downstream of the blockage.

### Extent of lung disease is classified using local pulmonary function measures

**Figure 4:**
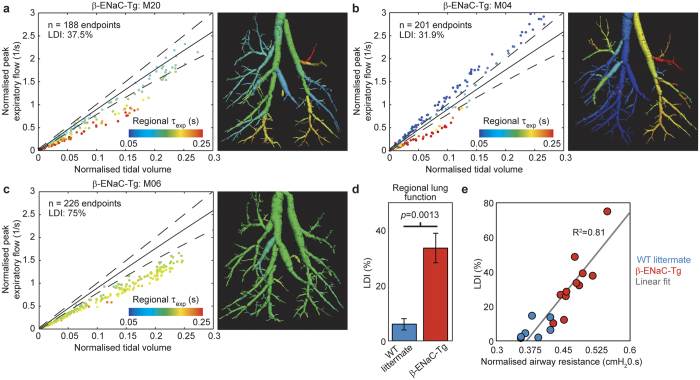
4DPIV followed by ATL provides regional quantification of the distribution of disease. (**a–c)** Scatterplots of normalised local peak expiratory flow against normalised tidal volume at endpoints in segmented airway tree for three β-ENaC-Tg mice. (**d**) Lung disease index (LDI), a parameter that utilises local pulmonary function data to classify the extent of lung disease; of WT littermate control and β-ENaC-Tg mice as measured using the analysis methods presented in this paper, indicate greater extent of lung disease in β-ENaC-Tg mice over WT controls. (**e**) LDI versus specific airway resistance measures from pulmonary function testing for WT littermate control (blue circles) and β-ENaC-Tg mice (red circles), including line of best fit (solid black).

Scatterplots of normalised peak expiratory flow versus normalised tidal volume at the endpoints of the segmented airway tree, depict the variability of lung disease affecting the β-ENaC-Tg mice (Fig. 4a–c). Figure 4a shows disease that is highly heterogeneous consistent with mucus blockages in the peripheral airways. Figure 4b shows a case where the entire left lung is affected by a blockage in the upper bronchi, and Fig. 4c shows a case where both lungs are affected by an obstruction in the trachea.

Lung tissue expansion is the direct result of the air flowing through the airways, into and out of the tissue. As such, lung expansion as measured using the methods described here, is a direct consequence of lung ventilation, the movement of air in to and out of the lungs via inhalation and exhalation. In this study, we use the terms ‘lung expansion’ and ‘lung ventilation’ interchangeably as they are physiologically related. We use measures for regional airflow (local measures for tidal volume, peak expiratory flow and expiratory time constant for regional airflow) at the endpoints to determine regions of tissue within the lung that are functioning below that observed in healthy littermates. Each endpoint feeds a certain volume of lung tissue (see Supplementary Fig. 2) and therefore regional analysis on each mouse provides a wealth of information. Here we introduce a parameter termed lung disease index (LDI), to reduce this data to a single index. The LDI indicates the percentage of total lung volume that is functioning below (i.e. a lower local peak expiratory flow) the healthy littermate group (see Methods), therefore quantifying the extent of disease within the overall lung. LDI was calculated and shown to be higher in β-ENaC-Tg mice over WT littermate controls (Fig. 4c). LDI measures for all mice also correlate to the airway resistance function measures obtained from FOT (Fig. 4d). Supplementary Fig. 3 shows the airway trees coloured by expiratory time constant for all mice in the study. Supplementary Fig. 4 shows additional scatter plots of normalised peak expiratory flow versus normalised tidal volume.

### Distribution of local expiratory time constant measures reveal greater insight to lung function over global measures

**Figure 5:**
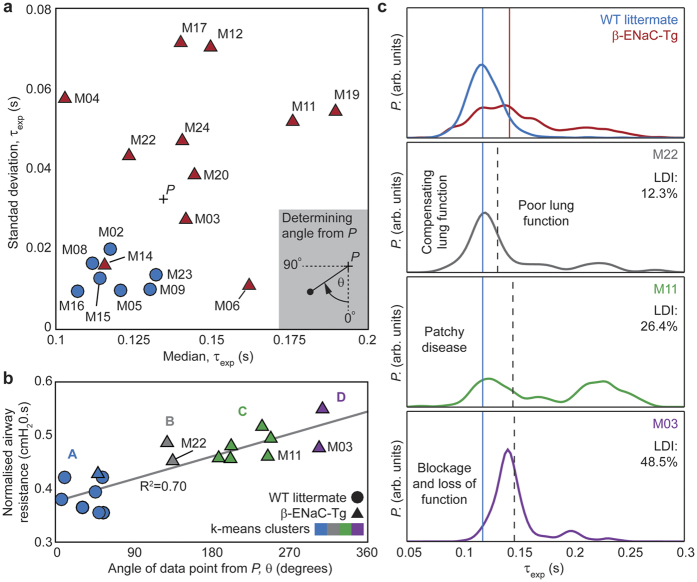
Distribution of time constant reveals severity of disease. (**a**) Standard deviation of expiratory time constant at endpoints versus median value of expiratory time constant at endpoints for WT littermate controls (blue circles) and β-ENaC-Tg mice (red triangles). Point, *P*, represents the intersection of the gross population’s mean values for median and standard deviation of expiratory time constant. (**b**) Specific airway resistance, as measured by pulmonary function test (flexiVent), plotted against the angle of each data point from point *P* in (**a)**. Data points are clustered into 4 groups using the k-means algorithm, based on distribution of expiratory time constant in (**a**). (**c**) Averaged histograms of expiratory time constant at endpoints from WT littermate and β-ENaC-Tg populations (top panel) and histograms of expiratory time constant for three β-ENaC-Tg mice that have similar pulmonary function test results (bottom panels). The blue solid line in each inset shows the average time constant from entire littermate population from the top panel. The black dotted line shows the averaged expiratory time constant at the endpoints for each individual mouse. While function test results are similar, our method shows 3 distinct phenotypes of pathology.

The distribution of expiratory time constants, measured at the endpoints obtained from 4DPIV followed by ATL (see Methods), was further explored to quantify the spatial variation in lung disease across the mouse sample populations used in this study (Fig. 5). A plot of the median against the standard deviation of expiratory time constant distributions for all mice in the study (Fig. 5a), depicts clear distinction between the closely grouped WT littermates and the widespread β-ENaC-Tg mice, indicative of the heterogeneity in lung function across each group. The point, P, denotes the average for the median and standard deviation expiratory time constant measures shown on the plot. Pulmonary function test results are plotted against the angle of each point on the plot from point P to differentiate between disease phenotypes (Fig. 5a-inset, Fig. 5b). The circles represent littermates and triangles denote β-ENaC-Tg mice. The colours represent the clusters into which these points are automatically grouped, using a k-means clustering algorithm^25^ implemented in MATLAB. K-means clustering is a popular method for cluster analysis in data mining and involve partitioning the data in to separate groups based on their statistics. Averaged histograms of expiratory time constants (individual data shown in Supplementary Fig. 5) are shown overlaid for all WT littermates and β-ENaC-Tg mice (Fig. 5c top) as well as three individual β-ENaC-Tg mice from each cluster (clusters B through D, Fig. 5c bottom three panels) selected using the k-means clustering algorithm on the basis that they exhibit similar airway resistance as measured using FOT (data points annotated in Fig. 5b). The blue line and red line (in top panel only) represent the mean expiratory time constants calculated for the WT littermate and β-ENaC-Tg mouse populations, respectively. The dashed black line represents the average expiratory time constant calculated for each individual mouse and is a close proxy to a global measure. This analysis reveals that our regional function measurement technique fully captures the spatial distribution of lung disease over current clinical assessment techniques that provide only a global measure for lung function. Supplementary Fig. 5 shows the individual histograms for WT littermate and β-ENaC-Tg mice that contribute to the averaged histograms in the top panel of Fig. 5c.

## Discussion

Here, we apply 4DPIV followed by ATL to quantify the heterogeneity of lung disease in βENaC-Tg mice and compare disease distribution and severity to their WT littermates *in vivo*. The βENaC-Tg mouse is a well-established model with patchy muco-obstructive lung disease characteristic of CF lung disease in humans.

Regional lung ventilation is determined from 4D lung motion analysis (4DPIV) and associated with the airway structure (ATL) to determine local airflow at each branch in the segmented airway tree^21^, allowing for detailed visualization of disease heterogeneity. Furthermore, our analysis revealed that a wealth of specific regional information on lung mechanics could be captured in addition to what can be provided by conventional global measurements.

Physiological lung function measures obtained from forced-oscillation testing (FOT) show that β-ENaC-Tg mice have significantly higher (*p* < 0.0001) specific airway resistance compared to their WT littermates. However, the airway resistance measured using spirometry is a global measure with major contributions from the conducting airways. Therefore, the dynamics of the lung tissue, as well as the spatial distribution and severity of the disease cannot be captured using FOT on existing lung function assessment methods. Expiratory time constants calculated from tidal volume data obtained from the proposed ATL analysis method shows a significant increase of airway resistance among β-ENaC-Tg mice, confirming the presence of significant lung disease. This finding is consistent with previous reports of the muco-obstructive pulmonary phenotype of βENaC-Tg mice, including substantial pulmonary mortality (45% in this study) in the first weeks of life^22^,^23^,^24^. Wongviriyawong *et al*.^26^ utilized a similar approach using PET imaging and airway tree morphology from high-resolution CT images, combined with a simple electrical analogy model of the airways to deduce regional resistance for lung segments in humans. In contrast, our method can provide dynamic data across the breath, allowing time-constant data to be calculated directly, which is not possible using a static PET image.

The volumetric analysis shown in this study extends previous work using 2D lung motion measurements of mice after bleomycin treatment^20^, which showed that regional lung expansion was a sensitive indicator of regional lung disease. Dubsky *et al*.^21^ applied this volumetric technique to healthy mice, and rabbit neonates. Here we combine and improve on these methods to quantify changes resulting from lung disease.

We show here, for the first time, a detailed and 3D view of the regional nature of lung function in β-ENaC-Tg mice. Airway mucus obstruction, typical in CF lung disease, produces higher resistances in the downstream airways. Histological observations validated the presence of mucus obstructions inferred from our analysis. Other parameters introduced in literature^27^,^28^,^29^ to capture lung disease heterogeneity do not properly describe the measure we can now provide. Therefore, we have introduced a new parameter, the lung disease index (LDI), derived from measures for regional ventilation, to quantify inhomogeneous lung disease.

Histogram analyses of expiratory time constants calculated at all the endpoints in the segmented airway tree shed further insight on the different phenotypes of lung disease in β-ENaC-Tg mice. WT littermate controls exhibit (see Supplementary Fig. 5) homogenous expiratory time constant (μ = 0.12s, σ = 0.022s) while the distribution for the β-ENaC-Tg population in the study is bi-modal (μ_1_ = 0.13s, σ_1_ = 0.038s; μ_2_ = 0.22s, σ_2_ = 0.037s). Individual data sets were categorized into four distinct groups based on distribution parameters. There was good agreement (R^2^ = 0.7) between the categories and normalised airway resistance from FOT analysis. One β-ENaC-Tg (M14) was incorrectly categorized by this conventional FOT analysis as a healthy mouse. We showed here that a global FOT measurement could not reveal the large variations in lung disease that were captured by 4DPIV followed by ATL. A bi-modal distribution of lung function, as shown in our results, has been previously demonstrated in other conditions such as asthma^30^, in which heterogeneity is potentially exacerbated by interdependence effects between lung regions^31^. Our identification of bi-modal lung function in β-ENaC-Tg mice, similar to those in a variety of lung conditions reveals a strength of our technique, and highlights the importance of imaging for assessment of lung disease generally, as global measures by definition must result in the loss of information on regional differences in the presence of disease.

In conclusion, 4DCT in conjunction with lung motion measurement (4DPIV) followed by ATL, was used to successfully quantify regional airflow in the airways of β-ENaC-Tg mice. We used conventional FOT to show that WT littermates could be distinguished from β-ENaC-Tg mice using measures for specific airway resistance, and to act as a method to validate our new lung disease index (LDI) that uses local expiratory time constant of airflow to quantify regional lung function. Here, we have shown regional lung function allows the detection and quantification of disease that is not available using global lung function tests such as spirometry or FOT. Our results suggest that this extra lung function information could aid early diagnosis of lung disease.

Although there is some debate about the significance of medically-delivered radiation^32^, the use of 4DCT could significantly limit the clinical utility of using lung motion to derive a measure for lung function. Alternatively, 4DPIV can be substituted with Computed Tomographic X-Ray Velocimetry (CTXV)^33^, an alternative low-dose flow (motion) measurement technique that can reconstruct the flow through an opaque vessel from as few as three projections^34^. Thereby CTXV followed by ATL would uniquely allow regional lung function mapping with much lower radiation dose than is delivered by 4DCT, which generally requires hundreds of projections (per time-point) for a good reconstruction. This reduced dose would make CTXV followed by ATL clinically applicable with the capability of delivering results similar to those in this study.

Earlier and more accurate disease detection is the key to pro-active interventions with the ability to limit lung disease progression. Additionally, sensitive assessment of the progression of disease or subtle reversal of disease in response to treatment is critical information that physicians require for successful management of disease. Measurements of lung motion and associated analyses deliver rich functional information that could now be used in conjunction with other methods for early diagnosis and management to achieve better patient outcomes and accelerate the development of new treatments.

## Methods

### Ethics statement

Experiments were performed at the third generation SPring-8 Synchrotron Radiation Facility in Hyogo Prefecture, Japan. The experimental procedures were approved by the Animal Care and Use Committees of the relevant institutions. Animal Ethics were approved by the Women’s and Children’s Health Network Animal Ethics Committee, Adelaide, South Australia. All the experiments were performed in accordance with the approved guidelines and regulations.

### Experimental animals

The β-ENaC mice (*n* = 11), aged between 44–57 days (median = 49 days) at time of imaging, originated from a mixed genetic background (C3H/HeN x C57Bl/6N bred with male C3B6F1 wild-type) breeding colony (University of Heidelberg, Germany). Littermate controls (*n* = 7) were used to eliminate the possible effects of strain background on lung phenotype. Offspring were genotyped at 3 weeks of age via PCR of genomic DNA as previously described^22^. The mice were bred and housed in a pathogen-free environment. Mice were anaesthetised by sodium pentobarbital (Somnopentil, Pitman-Moore, Washington Crossing, USA) i.p. injection, and non-surgically intubated for attachment to a computer-controlled custom-built small animal pressure-controlled ventilator (4Dx Limited, Australia); at 12 cmH_2_O PIP and 2 cmH_2_O PEEP with a respiratory rate of 133 breaths per minute (each cycle lasting 0.45 seconds with an inspiration time of 0.15 seconds and an expiration time of 0.3 seconds). Mice were mounted vertically in a custom rotational stage rig for imaging. Radiation dose was minimised by using a mechanical shutter synchronised to open only when images were being captured. Rectal temperature and ECG were monitored throughout the experiments. To preserve the normal dynamics of lung function, a paralytic was not administered.

### Phase contrast X-ray imaging (PCXI)

All PCXI was performed on the BL20B2 bending magnet beamline, with imaging performed in Experimental hutch 3 located in the Biomedical Imaging Center. A simplified experimental setup schematic is shown in Fig. 1a. The broadband synchrotron radiation was filtered by a Si-111 double crystal monochromator, providing 28 keV of beam energy, with a sample to detector distance of 3 m. Images were acquired using a modified beam monitor (AA50, Hamamatsu, Japan) and a sCMOS digital detector (pco.edge, PCO Imaging, Germany) with an effective pixel size of 15.3 μm. Images were captured at 37 frames per second (at an exposure time of 17 ms with a 10 ms delay between exposures) and synchronized with mechanical ventilation to acquire 16 frames per respiratory cycle. The short exposure time was used to reduce motion blur. The mice were imaged upright and 6500 images were captured per data set over an arc of 180 degrees.

### Pulmonary function measurement

Measurements of lung mechanics were performed in anaesthetised mice directly after X-ray imaging, using a computer controlled flexiVent (Scireq, Montreal, Canada) small animal ventilator to determine maximal vital capacity, airway resistance, dynamic compliance, tissue damping, tissue elasticity and hysteresivity. Airway resistance was divided by vital capacity to yield normalised airway resistance.

### Histology

Animals were humanely killed with a lethal dose of sodium pentobarbital after functional imaging and pulmonary function testing. The lungs were excised and fixed in 10% formalin for histology. Paraffin-embedded 5 μm sections were stained with AB/PAS.

### 4D X-ray lung velocimetry (4DPIV) and airflow calculation using Airway Tree Link (ATL)

Image acquisition was synchronized with mechanical ventilation, providing known and corresponding time-points throughout the respiratory cycle for all animals. Images were pre-processed using a flat-dark correction and a single-image phase retrieval routine^35^. The pre-processed projections were reconstructed using an algebraic tomographic reconstruction to obtain 3D volumes of data at 16 time-points along the respiratory cycle. Fig. 1b (I) shows the segmented lungs and ribs from a reconstruction at a single time-point. A 3-dimensional (3D) cross-correlation based motion measurement technique was used to obtain a 3D map of the lung displacement between two successive volumes in time (Fig. 1b (II)). The cross-correlation analysis was performed with interrogation regions of 64 × 64 × 64 voxels (representing a 980 μm^3^ region) with a regular spacing of 16 voxels between the centres of adjacent interrogation regions. Analysis on 16 reconstructed volumes resulted in 16 3D displacement fields (last volume correlated with the first volume along the resp. cycle) per animal. The expansion field of lung tissue was calculated from the local gradients in the lung displacement and is shown for a single time-point in Fig. 1b (III). The segmented airway tree (see Fig. 1b (IV) and methods: airway tree segmentation) was associated with the lung expansion maps to calculate the time-varying airflow throughout the bronchial tree using the Airway Tree Link (ATL) analysis method previously described in Dubsky *et al*.^21^. The ATL technique utilises the local ventilation values of lung tissue to directly infer the local airflow through each airway supplying a given region of tissue. A supplying airway, or ‘endpoint’, is the most distal segment in the segmented airway tree; that is, a segment in the airway structure that has a parent segment but no daughter segments. Assuming negligible compressibility effects, the principle of continuity dictates that at each bifurcation, the flow through a parent segment must equal the sum of the flow through its daughter segments. As the flow in each endpoint is known, the flow through the entire tree can therefore be calculated by recursively summing the airflows in daughter segments at each bifurcation to calculate the airflow through the parent segment.

### Airway tree segmentation

The airway tree was segmented from the CT volume at peak expiration. A vessel enhancing filter or ‘vesselness’ filter^36^ was applied to the CT volume. This filter produces a volume for the ‘vesselness’ parameter; a measure relating to the likelihood that any given pixel belongs to a tubular structure. The airway tree was segmented using a flood fill segmentation method in Avizo 3D Software (FEI Visualization Sciences Group) on the MASSIVE supercomputing environment at Monash University. The binary-segmented volume was skeletonized to obtain 3D topological data necessary for association with the lung tissue ventilation map and subsequent calculation of regional airflow.

### Expiratory time constant calculation

The expiratory time constant, τ_exp_, a standard measure for studying respiratory mechanics, contains information about the mechanical properties of the lung such as the resistance and compliance. τ_exp_ is often expressed as the RC time constant where R is the resistance of the system and C its compliance^37^. While τ_exp_ is a measure of the time required to empty the lungs, increased *τ*_*e*_ is indicative of airway obstruction and/or reduced lung recoil. Since the flow waveform (16 time-points) was controlled, integration of the flow waveform produced known volume waveforms. The expiratory time constant was then measurable at each branch of the airway tree, with τ_exp_ expressed in seconds, representative of the time required for the local volume in an airway to reach 63% of its tidal volume.

### Endpoint analysis and lung disease index

For each mouse in this study, a scatterplot (Fig. 2c) was created to show normalised local peak expiratory flow (local peak expiratory flow divided by local tidal volume at that endpoint) against normalised tidal volume (local tidal volume divided by volume at end expiration) at the endpoints in the airway structure. To obtain the behaviour of healthy mice, data from endpoints in the entire littermate control population (*n* = 7 mice, *n* = 1615 endpoints) was plotted (Fig. 2d left panel). Each point on the scatterplot represents a regional measure for lung function at each endpoint of the airway tree and are coloured by local expiratory time constant. A polynomial fit to littermate data was added (solid line) with 99% confidence intervals (CI; dotted lines). For any given data set, endpoints that correspond to lower peak expiratory flows than the 99% CI of the WT littermate data (see Supplementary Fig. 2), are considered to have air supplied from stiffer portions of lung tissue, i.e. diseased lung. Here we define a descriptor, the lung disease index (LDI), based on our regional measures for lung function. LDI indicates the percentage of total lung volume that is functioning below that observed in the WT littermate group, and allows to quantify the extent of lung disease between the two groups.

### Statistics

Unpaired two-tailed t-tests were performed to compare normalised airway resistance, average expiratory time constants and disease indices arising from 4DPIV analysis. Results were considered statistically significant if *p* < 0.05. Values were reported as mean ± SEM unless stated otherwise.

## Additional Information

**How to cite this article**: Stahr, C. S. *et al*. Quantification of heterogeneity in lung disease with image-based pulmonary function testing. *Sci. Rep.* **6**, 29438; doi: 10.1038/srep29438 (2016).

## Supplementary Material

Supplementary Information
