# Supplementary material for: Quantification of heterogeneity in lung disease with image-based pulmonary function testing
Source: Sci Rep. 2016 Jul 27;6:29438. doi: 10.1038/srep29438 (PMC4962033; doi:10.1038/srep29438)
Supplement: Supplementary Information [file srep29438-s1.pdf]

Quantification of heterogeneity in lung disease with image-based pulmonary function testing

Charlene S. Stahr, Chaminda R. Samarage, David W. Parsons, Martin Donnelley, Nigel Farrow, Kaye S. Morgan, Graeme Zosky, Richard C. Boucher, Karen K. W. Siu, Marcus A. Mall, Stephen Dubsky, Andreas Fouras

Supplementary Figures

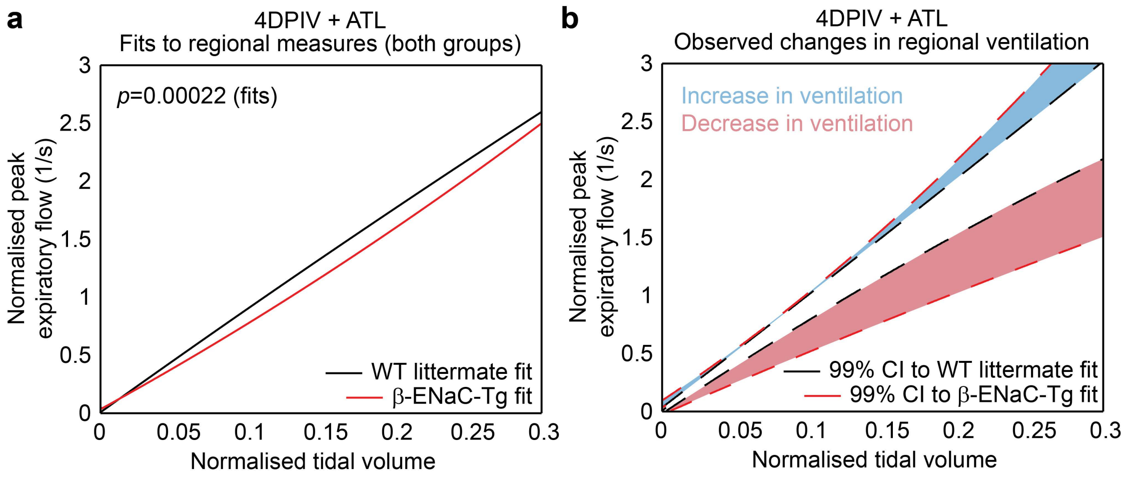

Supplementary Figure 1. Observed global changes to ventilation as a result of lung disease.

a, Polynomial fits (black line: WT littermate, red line: β-ENaC-Tg mice) to regional measures shown in Fig. 2d show there is a significant difference between regional measures obtained for the two groups.

b, 99% confidence intervals to polynomial fits of both groups (black line: WT littermate, red line: β-ENaC-Tg mice) highlight the existence of regions affected by lung disease. Significant reduction in airflow leads to a decrease in ventilation (red region) and increased expiratory time constants (data shown in Fig. 2d) in β-ENaC-Tg mice. However, an increase to ventilation (blue region), possibly as a sign of the lung compensating to maintain lung function, is also observed.

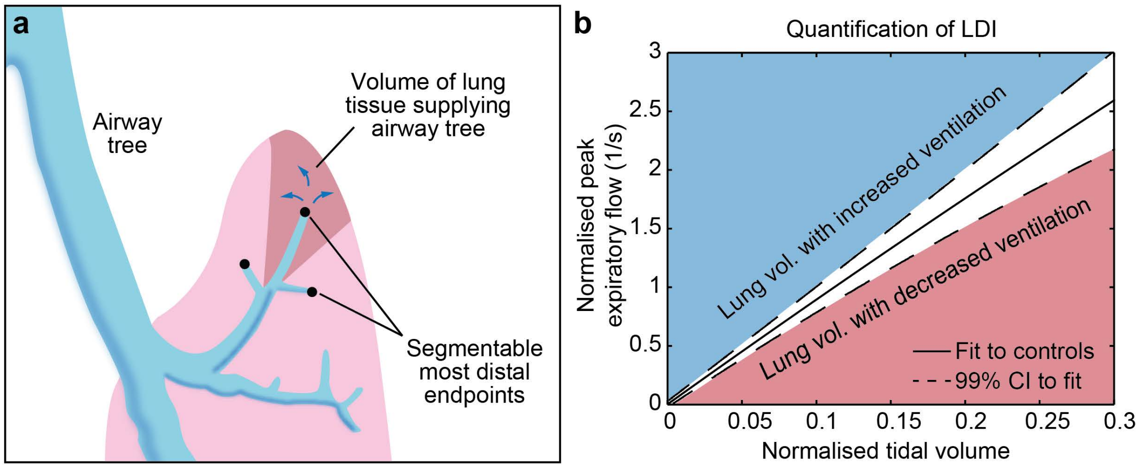

Supplementary Figure 2. Quantification of LDI.

a, Graphical representation of associating regions/volumes of lung tissue to the endpoints in the airway tree as used in the Airway Tree Link (ATL) process. The endpoints (black dots) are the most distal points segments in the airway tree that were segmented from CT. Each endpoint ventilates a volume of lung tissue and is the key parameter that is quantified by our lung disease index (LDI).

b, Graphical representation of the quantification of our lung disease index (LDI). Endpoint data of normalised peak expiratory flow is plotted against normalised tidal volume for all littermate controls. A polynomial fit and 99% confidence intervals (CI) are determined for entire littermate population used in this study. LDI is classified as the percentage of total lung volume corresponding to endpoints below the lower 99% CI interval, or regions with decreased ventilation in contrast to healthy littermates. This region is depicted here by the red region.

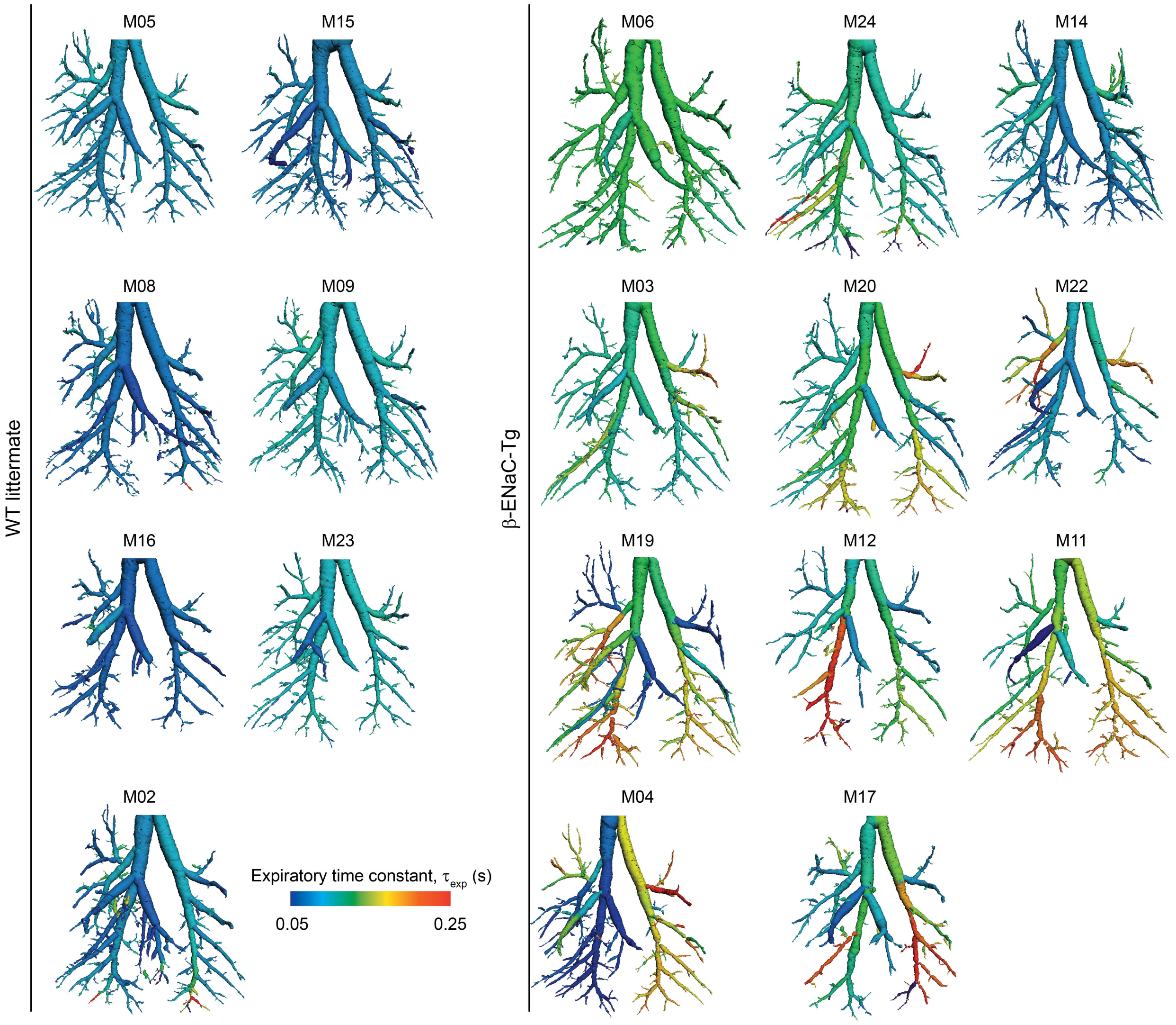

Supplementary Figure 3. Reconstructed airway trees for entire study population, 7 wild-type (WT) littermate control mice (left) and 11 β-ENaC-Tg mice (right), coloured by expiratory time constant. General uniformity of WT littermates and dispersed and variable expiratory time constant for β-ENaC-Tg mice corresponds to the known patchy nature of lung disease driven by airway obstruction caused by mucus plugging and limited clearance. Different regions of the lung are likely to compensate differently dependent on localized obstruction, and hence function and health of the lung.

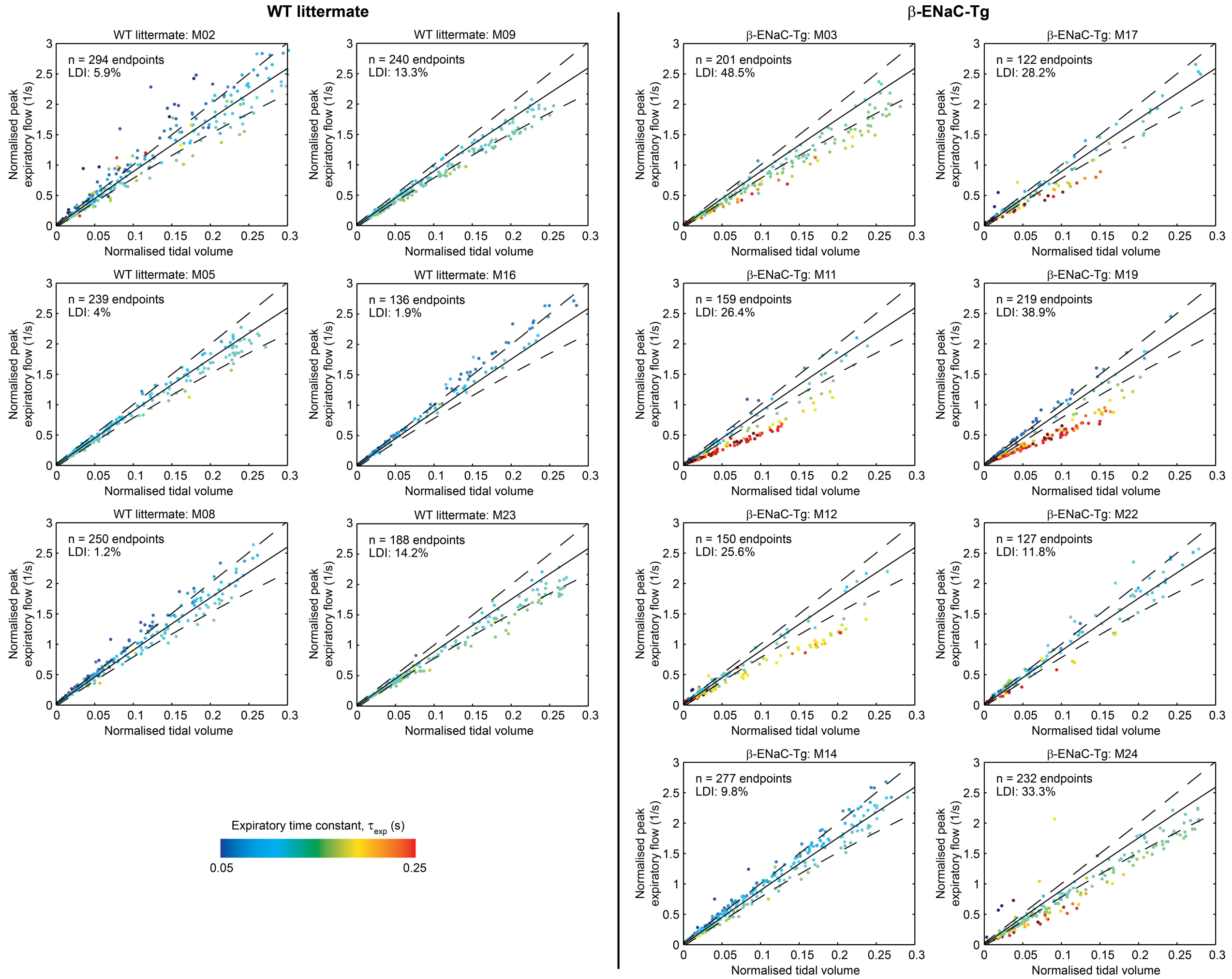

Supplementary Figure 4. Extended data from Fig. 4. Scatterplots of normalised peak expiratory flow against normalised lung tidal volume for all WT littermates (left) except M15 (shown in Fig. 2) and remaining β-ENaC-Tg mice data (right) not shown in Fig. 4.

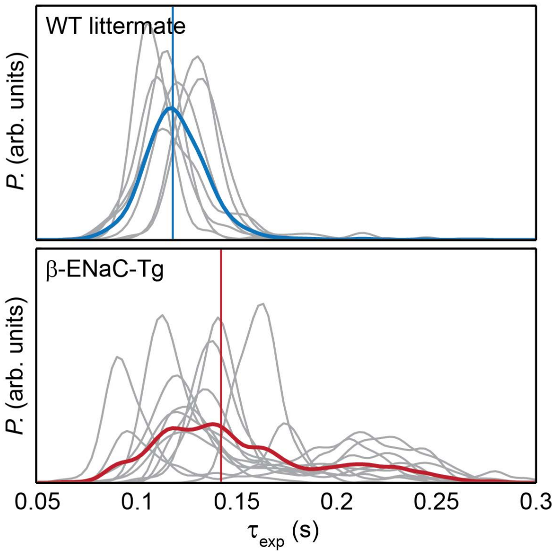

Supplementary Figure 5. Expiratory time constant histograms showing regional function distribution. Histograms of expiratory time constant distribution for each subject (grey), superimposed by average histogram for the WT littermate (blue, top) and β-ENaC-Tg mouse (red, bottom) populations used in this study.
